# Supplementary material for: Suppression of cGAS- and RIG-I-mediated innate immune signaling by Epstein-Barr virus deubiquitinase BPLF1
Source: PLoS Pathog. 2023 Feb 21;19(2):e1011186. doi: 10.1371/journal.ppat.1011186 (PMC9983872; doi:10.1371/journal.ppat.1011186)
Supplement: Attachment — Submitted filename: Reply to PP reviewer-17Dec.docx [file ppat.1011186.s005.docx]

**Point-to-point response to reviewers (PPATHOGENS-D-22-01354)**

Reviewer #1

1) The suppression of IFN-b production by BPLF1 is tested in artificial conditions such as overexpression of cGAS+STING, RIG-I N and TBK1. It would be nice to test the ability of BPLF1 to suppress signaling in response to a ligand, such as ISD90, triphosphorylated RNA, or polyI:C.

Response: We have tested the ability of BPLF1 to suppress cGAS-STING signaling stimulated by ISD90 in luciferase assay and immunoprecipitation experiments (S2 Fig). We employed HeLa cells for the Western blot experiments since it has intact cGAS-STING pathway. Upon ISD90 stimulation, we observed an increase in ubiquitination in total cell lysates as well as STING immunoprecipitates. When BPLF1 was expressed, ubiquitination levels in total cell lysates and STING immunoprecipitates drastically decreased, and the ubiquitination levels of both total cell lysates and STING immunoprecipitates partly resumed when BPLF1 was replaced by BPLF1-C61A. The biological effect is consistent with what we have shown with our overexpression experimental setting. For luciferase assay, we stimulated HEK293M81 cells with ISD90 and induced full-length BPLF1 expression with CRISPR-a system. We observed a decrease in relative luciferase activity when more guide 2 was introduced to the cells, indicating that the increase in full-length BPLF1 expression would mitigate ISD90-induced IRF3-Luc activity.

2) In Fig 3F, please add a control with no cGAS+STING expression. Also, the difference of the percentage of GFP positive cells is minor. Quantification by flow cytometry would be ideal.

Response: A control with no cGAS+STING has been added to Fig. 3F. We have also performed flow cytometry to quantify the percentage of GFP positive cells (Fig. 4). The result for wild type M81 virus infection in cells overexpressing different effectors is similar to our previous result.

3) In Fig 4 and 5, please add blot for WT and C61A BPLF1.

Response: Please find the expression of WT and C61A BPLF1 325 in Fig 3A.

4) In Fig4A, WT BPLF1 expression clearly reduced F-STING expression comparing lane 2 and 4. However, the author claimed that “The steady-state level of STING remained constant.” Although I agree that the perturbing the PTM of STING is more than likely to be the major effect of BPLF1, the author should be more careful about how to describe the data.

Response: We have modified the text to read “When BPLF1 was introduced into the cells, the ubiquitination of STING was diminished by more than 5-fold, even if normalized to the steady-state level of STING.”

5) Fig6C, how does BPLF1 perturb TBK1 PTM if it does not interact with TBK1? It is important to prove the interaction between TBK1 and BPLF1. Crosslinking the cell with DSG or DSP will be beneficial to capture transit protein-protein interactions.

Response: We have further performed endogenous TBK1 immunoprecipitation experiments with DSP crosslinking and still found no interaction between endogenous TBK1 and BPLF1. We have further discussed this to suggest that another adaptor may be required for BPLF1 to mediate its deubiquitinating activity on TBK1.

6) Line 342, the authors showed no evidence to support BPLF1 “interacted with the CMV promoter on the IRF3 plasmid vector to trigger IRF3 transcriptional activation.” Please modify the text.

Response: We have repeated the IRF3 dimerization experiment and updated Fig 7 to show that BPLF1 expression did not affect the IRF3 dimerization. We have modified our manuscript to describe BPLF1 mitigation of TBK1-induced IRF3 dimerization.

7) In Fig 9D, it seems like clone1 and clone 2 have extra bands at the top comparing to p2089. Please show uncropped figure.

Response: We have provided the full figure for your reference (see below). The extra bands at the top are some undigested products and would not affect genomic integrity of the mutant clones. As shown in the viral gene expression profile, latent EBV genes were expressed from the mutant clones and lytic genes were induced upon lytic reactivation treatment. These results showed that the mutant clones are functioning normally except having its BPLF1 deubiquitinase mutated.

Reviewer #2

1) Almost all work is done by overexpression. The results would be more relevant if some experiments could be confirmed with endogenous levels of proteins. Have attempts been made to examine protein interactions, ubiquitination state, and expression profiles in the absence of overexpressed TBK1, STING, etc?

Response: We are fully aware of the limitations of relying on overexpression in the study of the biological effects of BPLF1. However, only very few EBV-associated gastric carcinoma and NPC cell lines are available. We found it difficult to demonstrate the endogenous deubiquitination events of BPLF1 in physiologically relevant cell lines, particularly for the STING-IP Ub assays. We tried to conduct the experiments in AGS and YCCEL1 cells due to the ease of transfection. However, AGS and YCCEL1 do not have ample amount of endogenous STING protein for STING-IP. For NPC cell lines, they are very difficult to be transfected. Therefore, we turned to HeLa cells which express cGAS and STING for a model experiment of ISD90 stimulation. We observed ubiquitination of endogenous STING upon ISD90 stimulation and similar deubiquitination of STING when BPLF1 is present in HeLa cells (S2 Fig). The newly added results demonstrate that BPLF1 acts on endogenous protein like STING to mediate its deubiquitinating effect.

2) Additional BPLF1 expression does appear to result in increased infectivity, however it does no appear that additional C61A expression results in a “remarkable decrease” compared to mock as stated in line 235. This should be clarified.

Response: We have revised the text to read “no noticeable increase in the percentage of GFP^+^ cells was observed when C61A was expressed”.

3) If anything there appears to be a decrease in STING levels. Can the authors discuss this? Could changes in endogenous levels of STING be observed? Additionally in Figure 4B there is a prominent band just above the 34 kDa marker. Does this represent a ubiquitinated form of STING? I would expect a ubiquitinated form of STING to appear around 50KDa. Please clarify.

Response: We have revised the text to read “When BPLF1 was introduced into the cells, the ubiquitination of STING was diminished by more than 5-fold, even if normalized to the steady-state level of STING.” To further investigate this, in our supplementary figures for endogenous STING immunoprecipitations, we also observed a slight decrease in endogenous STING level, while the effect on deubiquitination of STING was much more pronounced.

For the prominent band above ‘34kDa’ marker, we believe we have mislabeled the protein ladder and the 34kDa mark should actually be 40kDa marker and the prominent band should be the monoubiquitinated form of STING with a molecular weight at around 55 kDa.

4) Could ubiquitination of TBK1 be observed without overexpression of TBK1? Both figure 4 and 5 would be more relevant if these could be shown with endogenous levels of STING and TBK1.

Response: We have performed the endogenous STING-IP experiments and we stimulated HeLa cells with ISD90 so that ubiquitinated STING can be observed (S1 Fig). The presence of BPLF1 would remove the ubiquitination on STING as shown in the Western blot of STING immunoprecipitates. The replacement of BPLF1 with BPLF1-C61A would restore ISD90-induced ubiquitination on STING1. This showed that BPLF1 can act on the ubiquitin moiety of endogenous STING.

5) Figure 6. IPs demonstrating interaction with BPLF1 and STING are convincing. Again, I would have liked to see this with endogenous levels. Was the opposite IP attempted to demonstrate an interaction between TBK1 and BPLF1?

Response: By several different approaches we were unable to see interaction between endogenous TBK1 and BPLF1. We have further discussed this to suggest that another adaptor may be required for BPLF1 to mediate its deubiquitinating activity on TBK1.

6) Figure 7. “...but I do have concerns and disagree with conclusions drawn in Figure 7…” “The authors state that BPLF1 mitigates TBK1-induced dimerization of IRF3 but this is not convincing to me from the data presented….” Address Figure 7 conclusions. Perhaps try to show effects of IRF3 dimerization in the absence of IRF3 overexpression or by other methods.

Response: We have repeated the IRF3 dimerization experiment and updated Fig 7 to show that BPLF1 expression did not affect the IRF3 dimerization. We have modified our manuscript to describe BPLF1 mitigation of TBK1-induced IRF3 dimerization. In the new figure, BPLF1 was found not to induce IRF3 expression but only affect the monomers, suggesting that unstimulated IRF3 could be observed when BPLF1 was expressed. Upon TBK1 overexpression, IRF3 dimer was observed. The TBK1-induced IRF3 dimerization would be significantly diminished in the presence of BPLF1, and only IRF3 monomer was observed. When BPLF1 was replaced with BPLF1-C61A, TBK1-induced IRF3 dimerization was remarkably restored.

7) NGS sequencing, detection of lytic gene products, or creation of a reversion would be helpful to establish that no unintended changes were introduced.

Response: We have detected lytic gene (Zta, Rta, BMRF2) expression for WT p2089 and mutant p2089 C61A clones and the expression levels of these lytic gene products are similar (S3 Fig). Both WT and mutant p2089C61A cells are just expressing a small amount of lytic EBV genes, indicating that the mutant virus is functioning normally with the mutation. We have also tried to induce lytic reactivation with TPA and sodium butyrate for both WT and mutant cell clones and both types of cells can be reactivated upon TPA/NaBt treatment as indicated with the increase in lytic gene products (Zta, Rta, BMRF2) expressions. Nanopore sequencing of the BACs has been conducted and no undesirable change was spotted in the mutant virus as mentioned in the legend to Fig 9.

8) In figure 10A there is much greater relative expression of C61A compared to WT BPLF1. Can the authors speculate why this may be?

Response: This may be due to prolonged culturing and adaption of the virus to the cellular environment. The p2089 virus in HEK293 cells have been adapted to the cellular environment for a longer while, which may lead to the methylation of the viral genome and decrease in viral gene expression, especially for the lytic genes. In contrast, the C61A mutant virus is relatively new to the cellular environment, so the viral genome is not as methylated as that of WT p2089, which would lead to more lytic gene expression. We have added some discussion on this issue.

Reviewer #3

1) However, the manuscript does require the inclusion of essential controls and preferably performing some key assays under more physiological conditions, as detailed below.

Response: Essential controls have been added and more experiments have been conducted under more physiological conditions.

2) The authors have included RIG-I inhibition by BPLF1 as a major point in the manuscript and title. Although RIG-I inhibition is likely due to the observed effects on TBK1 and IRF3 activation, besides a few assays in Figures 1-3, the majority of the assays in the remainder of the paper are focused on cGAS-STING. The authors should consider removing the emphasis on RIG-I, or include additional assays to strengthen this part of the manuscript.

Response: We were unable to add more figures, but we have decided to keep RIG-I in the title just to give a more balanced view on BPLF1.

3) Although most assays generally seem convincing, they often lack proper positive and/or negative controls or validation of protein expression levels. As indicated for the specific figure panels below, these should be included to confirm the validity of the assays.

Response: Requested controls have been added.

4) Performing a few key experiments in more physiological settings, for example by using exogenous stimuli or virus infection, would significantly strengthen the manuscript.

Response: We have added several experiments to enhance the physiological relevance of our study. For one example, to investigate the deubiquitination event in a more physiological setting, we stimulated the cells with ISD90 to induce ubiquitination on endogenous STING. We employed HeLa cells for these investigations since they have intact cGAS-STING-TBK1 signaling pathway. We found similar deubiquitinating event of BPLF1 on ISD90-induced ubiquitination of STING.

5) Furthermore, since the authors have prepared a BPLF1 C61A mutant virus, they could also perform infection experiments in wildtype and cGAS-deficient cells to determine the relevance of BPLF1-mediated cGAS-STING evasion during authentic infection.

Response: We have successfully produced mutant virus-producing cell lines. The cells were induced to produce BPLF1-C61A mutant virus upon Zta and gp110 transfection. We infected HEK293 cells overexpressing cGAS + STING or TBK1 with the mutant virus and observed a drastic decrease in infectivity compared to WT p2089 virus (S2 Fig). This decrease in infectivity for p2089C61A mutant virus was partially restored when BPLF1 was co-expressed. In addition, we found that upon cGAS and STING overexpression, cellular IFN-β mRNA level has markedly increased in cells infected with p2089C61A mutant virus, whereas the IFN-β mRNA level has not shown significant induction by WT p2089 virus. This piece of new data demonstrates the importance of the deubiquitinating ability of BPLF1 in EBV infectivity and the effect of the deubiquitinating effect of BPLF1 on cellular type I IFN induction by EBV.

6) Figure 1: The authors show two different doses of cotransfected BPLF1 in panels B-D. However, both doses are equally potent in suppressing IFNB-Luc. The authors could consider including a lower dose of BPLF1 to show a dose-dependent effect.

Response: We now transfected with a lower dose of BPLF1 as suggested.

7) Figure 2: The induction of luciferase activity by IRF3 in panels B and C is rather low (2 to 4-fold). The authors should comment on why this is the case and if that affects their ability to observe BPLF1-mediated inhibition of IFNB-luciferase induction. These assays would also become more reliable if Western blot analyses verifying BPLF1 levels were included.

Response: The relative low induction of luciferase activity by IRF3 is probably due to the relatively low expression level of the IRF3/IRF3-5D constructs. We have done the IRF3-Luc assay with IRF3-5D overexpression with increasing full-length BPLF1 expression using CRISPRa in HEK293M81 cells, and we found that the we can induce higher relative luciferase activities with IRF3-5D (~10 fold) and the increasing dosages of BPLF1-G2 would not lead to any suppression of IRF3-5D-induced relative IRF3-Luc activity as it did on cGAS-STING-, RIG-I N-, TBK1-induced IRF3-Luc.

8) Figure 2: In panel G, it cannot be ruled out that the CRISPRa approach leads to general EBV reactivation and lytic gene expression. To validate the specificity of their CRISPRa approach in upregulating only BPLF1, the authors need to compare the abundance of one or more other lytic EBV genes besides BPLF1 between the mock and guide-1/2 conditions.

Response: We have also tested other lytic EBV gene (Zta) to check whether CRISPRa leads to reactivation of EBV. We found no induction of lytic genes following CRISPRa treatment.

9) Figure 2: In panel H, the authors should include a control that is not affected by BPLF1 expression (e.g. IRF3 overexpression) to verify that sgRNA-cotransfection does not result in a non-specific reduction in IFNB-Luc activity.

Response: We have done that as per your suggestion and updated the figure accordingly.

10) Figure 2: In panel J, the authors conclude that the effects of NF-κB are not involved in their assay since they do not observe an inhibitory effect by IκB-sr. However, the authors need to show that they achieve functional IκB-sr expression, for example by including an NF-κB luciferase reporter whose induction is expected to be inhibited by IkB-sr.

Response: We have now shown this in S1C Fig.

11) Figure 3: Since the differences in GFP expression in panel F are relatively small, it is hard to quantitatively assess these results. The authors should include a quantification of the percentage of GFP+ (infected) cells, for example by flow cytometry. Especially since the difference in EBV DNA content in panel G is also relatively minor (1.5 fold increase). Furthermore, to allow appreciation of the effect of cGAS/STING and TBK1 overexpression on virus infection efficiency, the authors should also include a sample without overexpression of these signaling molecules.

Response: We have performed flow cytometry to better quantify the percentage of GFP positive cells and updated the figure accordingly with the mock infection group. The result for WT M81 virus infection in cells overexpressing different effectors is similar to our previous result.

We were also able to produce the mutant p2089-C61A virus for viral infection study. We employed the p2089-C61A virus for primary EBV infection in cells overexpressing cGAS and STING and TBK1. We found that the p2089-C61A virus showed much lower infectivity than the mock infection group. When we co-expressed BPLF1 with cGAS and STING, as well as TBK1, the infectivity of the mutant p2089-C61A virus was partially restored (S5 Fig). These two pieces of data can demonstrate that the deubiquitinating ability of BPLF1 is crucial for EBV infectivity.

12) Figure 4: In panel A, the authors should include a Western blot showing successful and equal precipitation of FLAG-STING in each of the samples (IP: FLAG, WB: FLAG).

Response: We noticed the slight decrease of FLAG-STING when BPLF1 was expressed. Regarding the drastic deubiquitinating event on STING in the presence of BPLF1, we believed that this slight decrease would not alter the interpretation of BPLF1-mediated physiological effect on STING.

13) Figure 4: For panels B and C, the images are partly overexposed and cannot be interpreted. The authors need to (additionally) include lower exposures of these blots.

Response: We tried to capture the ubiquitination levels of different kinds of ubiquitin moieties (WT, K48, K63, K27, K48R, K63R) in the same panel. The WT Ub group would usually have much stronger ubiquitin ladders than the other groups so the ubiquitination of WT Ub group may be a bit overexposed. However, we believe that it would not affect our interpretation on the deubiquitination ability of BPLF1. The presence of BPLF1 would significantly reduce the ubiquitination levels in the WT Ub group and the replacement of BPLF1 to BPLF1-C61A would restore the ubiquitination. Shorter exposure of key panel has been added.

14) Figure 5: Some images cannot be interpreted due to overexposure. The authors need to include (additional) lower exposures.

Response: The chosen images can capture the biological effect of BPLF1 on different ubiquitin moieties. Short exposure of the key panel has also been provided.

15) Figure 7: The authors show that BPLF1 expression enhances ectopic IRF3 expression, which they conclude is mediated by the CMV promoter. Can the authors comment on whether they have additional evidence for the involvement of the CMV promoter, and whether this effect affects other assays presented in the manuscript, e.g. the luciferase assays?

Response: We have repeated the IRF3 dimerization assay and found no induction of IRF3 dimerization in the presence of BPLF1. We have modified the text accordingly.

16) Figure 8: In panel C, the authors compare BPLF1 and LMP1 expression and conclude that AGS-BX1 cells express relatively high levels of BPLF1. Since LMP1 is a latent transcript, the relative increased expression of BPLF1 could mean that there is more BPLF1 in latently infected cells, or that a higher percentage of AGS-BX1 cells is undergoing (spontaneous) lytic reactivation in comparison to the other cell lines. The authors should comment on this.

Response: As discussed in the text, AGS-BX1 might undergo spontaneous lytic reactivation since low levels of Zta and Rta mRNA were detected without any treatment with lytic inducer.

17) Figure 8: In panel F, the authors should consider including a non-targeting control sgRNA to show specificity of the observed inhibition of IFN mRNA induction by the BPLF1-targeting sgRNAs.

Response: We have checked the EBV gene expression profile and no other EBV gene was found to be induced upon CRISPRa treatment.

18) Figure 10: As acknowledged by the authors in the Discussion section, clonal differences between the p2089 and p2089-C61A variants may cause differences in the presented assays. Even though the authors have tried to diminish these effects by including several independent clones, a control should be included (e.g. IRF3 transfection) that is not affected by BPLF1 to verify that the signaling pathway leading to IFNB-Luc activation is similarly active in each set of clones.

Response: We have done the luciferase assay as per your suggestion and found no difference in IRF3-5D-induced relative luciferase activities in HEK293p2089 and HEK293p2089C61A cells.

19) Figure 10: Moreover, only a minor proportion of the cells is likely undergoing spontaneous lytic EBV infection and expresses BPLF1. The cGAS-STING pathway is thus expected to be induced primarily in cells not expressing BPLF1. The authors should comment on why they still observe a robust reduction in IFNB-Luc activity in the p2089 samples.

Response: BPLF1 is a potent deubiquitinase. In the p2089 cells, we detected notable amount of BPLF1 transcripts which indicates the presence of functional BPLF1. The expression of these spontaneously lytic BPLF1 may account for a major part of IFN-β production suppression since in the mutant C61A clones, the suppression on cGAS-STING-induced IFN-β production is minimal.
