# Supplementary material for: Suppression of cGAS- and RIG-I-mediated innate immune signaling by Epstein-Barr virus deubiquitinase BPLF1
Source: PLoS Pathog. 2023 Feb 21;19(2):e1011186. doi: 10.1371/journal.ppat.1011186 (PMC9983872; doi:10.1371/journal.ppat.1011186)
Supplement: Attachment — Submitted filename: Reply to PP reviewer-31Jan.docx [file ppat.1011186.s006.docx]

**Point-to-point response to reviewers (PPATHOGENS-D-22-01354R1)**

Reviewer #2

In point 7 of the rebuttal the authors show mRNA levels for ZTA, RTA, and BMRF2 (S3) and in their response state the levels are "similar." However ZTA mRNA levels look to be much higher with the C61 mutants. They are also somewhat higher with the BMRF2 mRNA. In Figure 10A there is a much greater relative expression of C61 compared to WT and the authors speculate this may be due changes occurring in culture over time and suggest differences in genome methylation. The unknown difference that may have evolved during passage in culture could also contribute to the observed phenotype. The difference in mRNA levels of ZTA and BMFRF2 should be mentioned in the text.

Response: We have modified the text to describe the difference in the levels of Zta and BMRF2 lytic transcripts in the text (lines 405-406). We have also added this to the discussion (lines 523-524 and 526-527).
